# Pursuit of hidden rules behind the irregularity of nano capillary lithography by hybrid intelligence

**DOI:** 10.1038/s41598-023-41022-7

**Published:** 2023-08-22

**Authors:** In Ho Cho, Myung Gi Ji, Jaeyoun Kim

**Affiliations:** 1 Department of Civil, Construction, and Environmental Engineering, Iowa State University, Ames, IA 50011 USA; 2 Department of Electrical and Computer Engineering, Iowa State University, Ames, IA 50011 USA

**Keywords:** Nanoscale devices, Computational science

## Abstract

Nature finds a way to leverage nanotextures to achieve desired functions. Recent advances in nanotechnologies endow fascinating multi-functionalities to nanotextures by modulating the nanopixel’s height. But nanoscale height control is a daunting task involving chemical and/or physical processes. As a facile, cost-effective, and potentially scalable remedy, the nanoscale capillary force lithography (CFL) receives notable attention. The key enabler is optical pre-modification of photopolymer’s characteristics via ultraviolet (UV) exposure. Still, the underlying physics of the nanoscale CFL is not well understood, and unexplained phenomena such as the “forbidden gap” in the nano capillary rise (unreachable height) abound. Due to the lack of large data, small length scales, and the absence of first principles, direct adoptions of machine learning or analytical approaches have been difficult. This paper proposes a hybrid intelligence approach in which both artificial and human intelligence coherently work together to unravel the hidden rules with small data. Our results show promising performance in identifying transparent, physics-retained rules of air diffusivity, dynamic viscosity, and surface tension, which collectively appear to explain the forbidden gap in the nanoscale CFL. This paper promotes synergistic collaborations of humans and AI for advancing nanotechnology and beyond.

## Introduction

**Figure 1:**
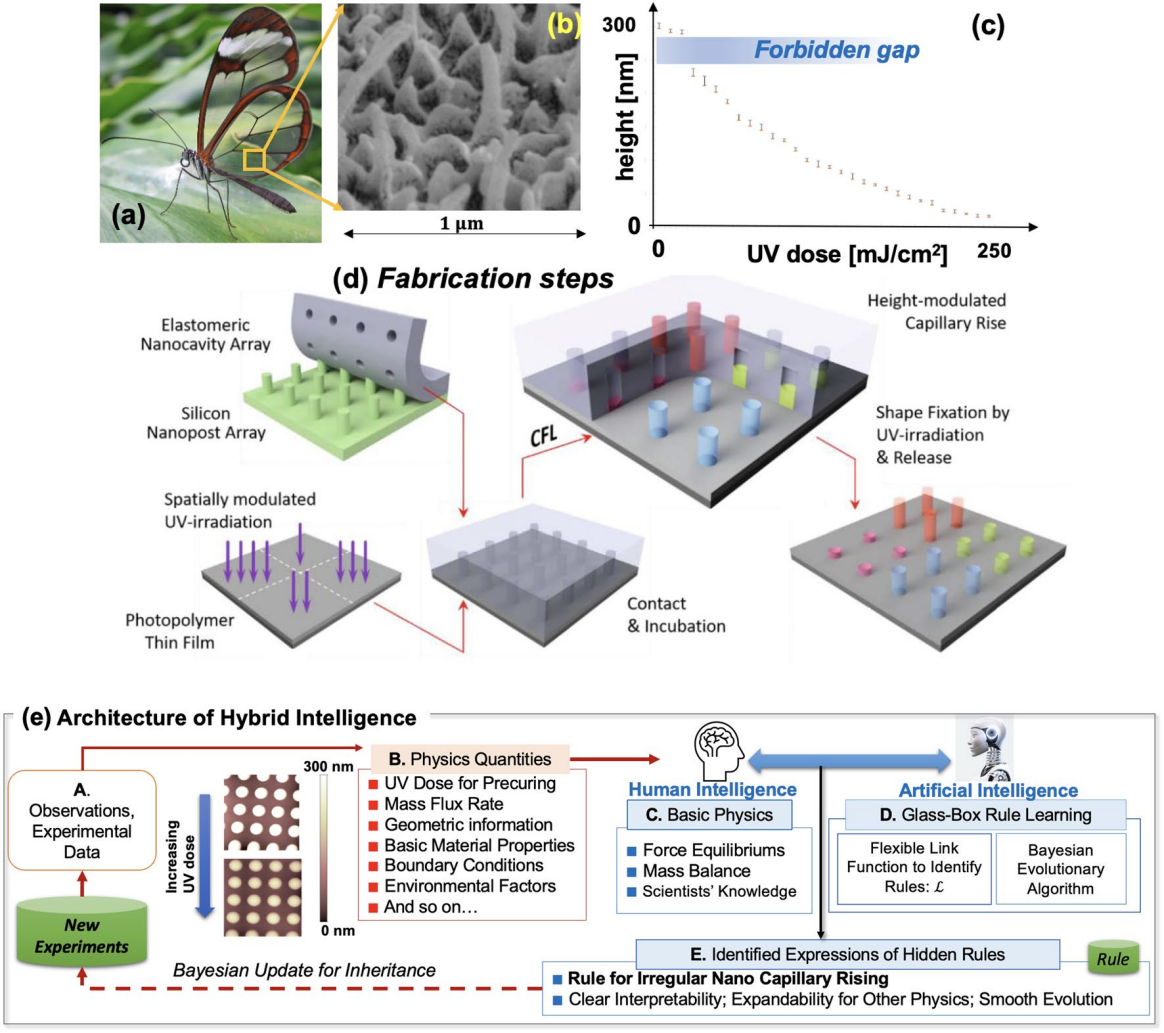
(**a**,**b**) Near-transparent glasswing of *Greta oto* and nanopillars of the wing (adapted from *wikipedia*); (**c**) Height plots obtained from the rise of the same photopolymer into cylindrical nanocavities. The data ranges were obtained from 10 measurements. The “forbidden gaps” is apparent near the low-UV dose regime; (**d**) Height-modulated nanopixel generation scheme; (**e**) Overall architecture of the proposed hybrid intelligence rule-learning framework. Step A shows the nano-scale experiments and observations. The insets are the real snapshots of the nanopillars made with varying UV doses. Step B summarizes basic physics features which function as the input features to the hybrid intelligence framework. Steps C and D correspond to the two cores of the hybrid intelligence framework—basic physics formulations by human intelligence (Step C) and transparent rule-learning methods (denoted “glass-box rule learning”) to unravel the hidden rules’ expressions (Step D). Step E stores the identified rules behind the irregular nano capillary rising which can be expanded with more physics and evolved with more data.

Nature finds a way to leverage nanotextures to achieve desired functions. For instance, the butterfly wing accommodates and modulates nanopillars (Fig. 1a,b) to achieve omnidirectional suppression of reflection as well as its beautiful colors. Recent advances in nanotechnologies endow fascinating multi-functionalities to nanotextures by modulating their nanopixel’s height distributions^1–5^. Nanoscale height control often requires daunting fabrication tasks involving chemical and/or physical processes^6–16^. As a facile, cost-effective, and potentially scalable remedy, the nanoscale capillary force lithography (CFL) receives notable attention, for which the key enabler is the optical premodification of the photopolymer’s characteristics via ultraviolet (UV) exposure^17^. Figure 1d summarizes the height-modulated nanopixel generation scheme using CFL (details of experiments are presented in Method). Despite the ease and general controllability, the underlying physics of the nanoscale CFL is not well understood. Experiments uncovered interesting yet unexplained phenomena such as the instability in the nano capillary rise, i.e., the abrupt jump in the nanocapillary rise, which leaves a “forbidden gap” in the nano capillary rise height (Fig. 1c) even under continuously varied UV doses^17^. New technology appears to call for new discoveries of hidden rules.

There have been many attempts to understand nanoscale phenomena related to this study. An analytical explanation was derived by^18^ for the fluid-filling process in a closed-end nanochannel. Theoretical investigations into capillary flow were conducted by^19^ for open microchannels with an evaporation effect while mathematical investigations were conducted by^20,21^ for the stability and continuous evolution of capillary surfaces in the so-called exotic cylinder (i.e., allowing entire equilibrium menisci by a varying radius). Due to the high compressibility of gas and the pressure-dependent effective permeability (denoted ${k}_{g}$), the Klinkenberg effect^22^ arises in porous media, resulting in ${k}_{g} \propto {k}_{\infty }$ and $b/p$. Here ${k}_{\infty }$ is the gas-phase permeability of the media under a high pressure, $p$ is the pressure, and $b$ is the Klinkenberg factor which depends on the pore structure and gas temperature. Many attempted to incorporate the Klinkenberg effect into the gas flow in porous media via analytical approach^23^, computational scheme^24^, and kinetic theory^25^. All of the prior research offers valuable insights and knowledge. This paper seeks to leverage them as basic physics on the “hybrid intelligence” framework to unravel the hidden rules underlying the process of nanoscale capillary force lithography.

### Hybrid intelligence framework for unraveling hidden rules of nano capillary lithography

This paper focuses on learning the hidden rules behind the intriguing behaviors of liquid-phase photopolymer rising into nanoscale capillaries, notably using the “hybrid intelligence” approach. The overall architecture of the hybrid intelligence approach is explained in Fig. 1e. Hybrid intelligence combines human and artificial intelligence. Human intelligence provides basic physics and scientists’ observations, experiences, and knowledge (Fig. 1e-Step C) while artificial intelligence offers computational and searching power to find the most promising expressions of the unknown rules (Fig. 1e-Step D). In the hybrid intelligence approach, the human’s insight and the machine’s computing power play in concert to unravel hidden rules.

The proposed approach is different from existing machine learning (ML) approaches and seeks to overcome their limitations. Direct adoptions of deep learning, reservoir computing^26^, or recurrent neural networks^27,28^ may predict the final values or mimic the final outputs successfully but cannot reveal the hidden rules of the system. Recent rule-learning ML methods^29–31^ show some promise, but they are based on known principles that were used for large data preparations/validations. Data- and ML-driven exploration of hidden mechanisms is in its infancy. In applying the hidden rule learning to the nanocapillary effect, the fundamental difficulty stems from the small length scale and time-varying salient properties such as the pressure, temperature, and mass, and their collective impacts on the diffusivity, dynamic viscosity, and surface tension. Obtaining reliable, informative large data sets is often difficult and expensive. In some cases, internal data are intrinsically inaccessible due to the technological limit of our generation, e.g., lack of a precise means to monitor the liquid’s behavior inside the nanocavity during the capillary rise. Direct adoption of black-box style ML methods may be unsuccessful.

The relative standing compared to existing statistical and ML approaches is noteworthy. The proposed hybrid intelligence approach is more general than statistical fitting methods using the least squares. As illustrated by Fig. S1, the hybrid intelligence helps systematically explore the diverse candidates of input-parameter pairs $x\to y$ (e.g., $x\in$ {UV dose, mass flux rate, …} and $y\in$ {diffusivity, viscosity, surface tension, …}) and helps find the most reasonable relations (i.e., shapes of the rule) of the pair. But detailed observations of $y$ are not available, rendering direct adoption of statistical fitting infeasible. Each of the internal parameters ($y$) can be regarded as a latent variable which is often learned by “encoder” ML methods^29–31^, with deep neural networks (Fig. S1e). Still, due to the small-sized data, direct adoption of encoder-based ML methods is not suitable here. As shown in preliminary investigations (Fig. S1f,g), extreme cases (i.e., too simple, or too complex rules) appear to fail to reach the desired accuracy, thus being rejected by the hybrid intelligence. Searching for the best parameters shares the central notion of the maximum likelihood method^32^.

The key strength of the hybrid intelligence approach lies in that it can learn hidden rules of multiple latent variables with a small-sized data set. The specific goal of this paper is to apply the hybrid intelligence approach to the hitherto unexplored irregularity of nano capillary lithography, the “forbidden gap” phenomenon. By combining physics principle-based formulations and ML’s rule-learning power on the hybrid intelligence framework, this paper seeks to offer a new interpretable answer, of which expansion shall be straightforward as new physics and more data become available in the future.

## Results

**Figure 2:**
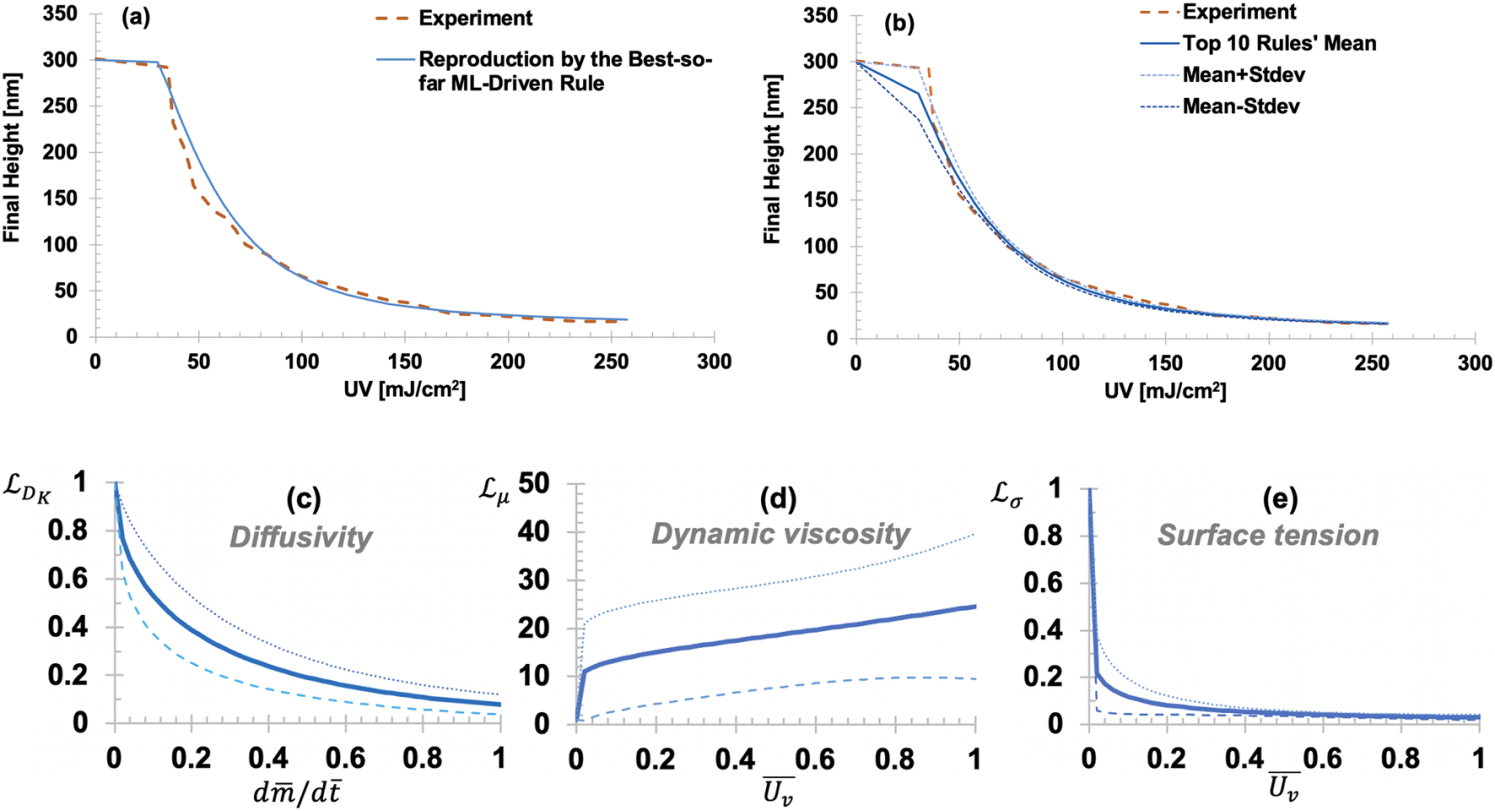
Best-so-far ML-identified rules: (**a**) Reproduction of the final height of nano capillary rising of UV pre-cured NOA73. Solid line shows the reproduction using the best-so-far ML-identified rules; (**b**) The mean and one standard deviation (Stdev) range of the reproductions using the top 10 ML-identified rules. In all cases, the sudden drop (called “forbidden gap”) experimentally observed near 35 $\mathrm{mJ}/{\mathrm{cm}}^{2}$ is well captured by the transparent ML-identified rules. Nanocapillary dimensions: diameter = 350 nm and height = 300 nm. (**c**) Identified link function (LF) of diffusivity of air molecules into the PDMS nanopores, ${\mathcal{L}}_{{D}_{K}}$($\frac{d\overline{m} }{d\overline{t} };{{\varvec{\uptheta}}}_{{D}_{K}}$) a function of the normalized air mass flux rate ($d\overline{m }/d\overline{t }$); (**d**) LF of the dynamic viscosity of NOA73 (pre-cured by UV), ${\mathcal{L}}_{\mu }$($\overline{{U }_{v}};{{\varvec{\uptheta}}}_{\mu }$) a function of the normalized UV dose ($\overline{{U }_{v}}$); (**e**) LF of the surface tension of NOA73 (pre-cured by UV) ${\mathcal{L}}_{\sigma }$($\overline{{U }_{v}};{{\varvec{\uptheta}}}_{\sigma }$). In (**c**–**e**), the dashed lines show the range of standard deviation of top 10 best-so-far rules. The best-so-far free parameters ${\varvec{\uptheta}}$ are presented in Table S1.

Figure 2a,b shows reproductions of the nano capillary rising heights by using the top 10 best-so-far rules. The transparent ML sought to learn three hidden rules (Fig. 2c–e). Two rules are about the dynamic viscosity (Fig. 2d) and surface tension (Fig. 2e) of the photopolymer (NOA73), as nonlinear functions of the total UV doses. The third rule is about the air diffusivity (Fig. 2c) as a nonlinear function of air mass flux rate into the nanopores in the mold made of PDMS. At the sudden change in the height (called a “forbidden gap”) near the UV dose at 35 $\mathrm{mJ}/{\mathrm{cm}}^{2}$, we conducted AFM analyses on the samples right above and below the forbidden gap for more quantitative comparisons. The obtained AFM images above and below the forbidden gap reveal that the final heights are 292 nm and 232 nm, respectively (details are shown in Fig. S7). By applying the glass-box ML-identified rules, the final heights right above and below the forbidden gap were well reproduced (Fig. 2a,b). The noticeable uncertainty in the rule of dynamic viscosity (Fig. 2d) shall be reduced by conducting Bayesian rule-learning with more data.

### Human intelligence for providing basic force equilibrium and mass balance

**Figure 3:**
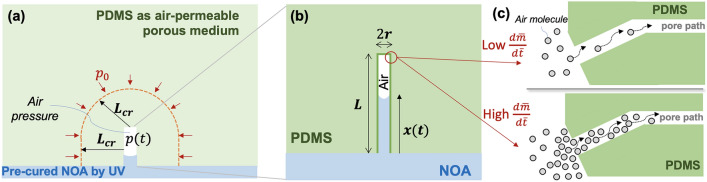
Pressure boundary conditions, geometry, and air mass flux during the capillary rise in a single nanocavity: (**a**) PDMS is regarded as an air-permeable porous medium. $p(t)$ is the time-varying pressure of the trapped air. The far pressure at the semi-spherical surface (marked by the dashed orange line, ${L}_{cr}$ [nm] away from the wall of the nanocavity) is assumed to be the atmospheric pressure ${p}_{0}$; (**b**) Basic geometry of a nanocavity accommodating the nano capillary rise of the UV pre-cured NOA73; (**c**) Illustration of two extreme cases of low and high air mass flux rates through the pore path of PDMS.

Detailed formulations and terms of basic physics-driven force equilibrium and mass balance of the nano capillary rise are presented in Method. Geometric terms are shown in Fig. 3. To obtain a concise analytical form and facile investigation, the rise distance, mass, and time are normalized as $\overline{x }=\frac{x}{L}\in {\mathbb{R}}[\mathrm{0,1}]; \overline{m }=\frac{m}{m\left(0\right)}\in {\mathbb{R}}[\mathrm{0,1}]; \overline{t }=\frac{t}{{t}_{rise}}{\in {\mathbb{R}}}^{+}$ where ${t}_{rise}$ [s] is the time required to fill the open-ended nanocavity (i.e., no pressure-dependent force term) calculated as ${t}_{rise}={\int }_{0}^{L}{\left(\frac{dx}{dt}\right)}^{-1}ds={\int }_{0}^{L}{\left(\frac{r\sigma ({U}_{v})\mathrm{cos}\left(\theta \right)}{4\mu ({U}_{v})s}\right)}^{-1}ds=\frac{2\mu ({U}_{v}){L}^{2}}{r\sigma ({U}_{v})\mathrm{cos}\left(\theta \right)}$. $\mu$ is the dynamic viscosity, ${U}_{v}$ is UV dose, $\sigma$ is the surface tension, and $\theta$ is the contact angle between NOA73 and the cavity wall. After normalization, we obtain the normalized equations of force equilibrium and mass balance as

1 $$\frac{d\overline{x} }{d\overline{t} }=\frac{1}{2\overline{x }\left(\overline{t }\right)}-\frac{\alpha }{2}\left(\frac{\overline{m }\left(\overline{t }\right)-1+\overline{x }\left(\overline{t }\right)}{\overline{x }\left(\overline{t }\right)\left(1-\overline{x }\left(\overline{t }\right)\right)}\right)$$

2 $$\frac{d\overline{m} }{d\overline{t} }=-\beta \left(1+\frac{2L}{r}\left(1-\overline{x }\left(\overline{t }\right)\right)\right)\left(\frac{\overline{m }\left(\overline{t }\right)-1+\overline{x }\left(\overline{t }\right)}{\left(1-\overline{x }\left(\overline{t }\right)\right)}\right)$$

where additional coefficients are introduced for brevity as $\alpha =\frac{\pi {r}^{2}{p}_{0}}{2\pi r \sigma ({U}_{v})\mathrm{cos}\left(\theta \right)}=\frac{r{p}_{0}}{2\sigma ({U}_{v})\mathrm{cos}\left(\theta \right)}$ meaning the ratio between atmospheric pressure and the Laplace stress. $\beta =\frac{2\mu \left({U}_{v}\right){D}_{K}\left(\frac{d\overline{m} }{d\overline{t} }\right)RT}{r\sigma ({U}_{v})\mathrm{cos}(\theta )}\frac{L}{{L}_{cr}}$ where ${D}_{K}\left(\frac{d\overline{m} }{d\overline{t} }\right)\equiv {D}_{d}{K}_{H}$ is a function of the air mass flux rate. ${D}_{d}$ is the diffusion coefficient of the air into PDMS mold. ${K}_{H}$ is the Henry constant determining the concentration of air molecules. ${L}_{cr}$ is the nanopore pressure critical length measured from the nanocavity’s wall. The rationale for ${L}_{cr}$ is presented in Method. $R$ is the universal gas constant, and $T$ is the absolute temperature. As a sudden increase in the traffic volume at a fixed intersection can cause a traffic jam, this study assumes that a sudden increase in the airflow into the PDMS nanopores can decrease the air diffusion of air into the PDMS nanopores. Figure 3c illustrates this physical rationale schematically. It is difficult to obtain accurate time-varying models of individual ${D}_{d}$ and ${K}_{H}$, separately. Rather, the combined effect of ${D}_{d}{K}_{H}$ (i.e., by ${D}_{K}$) is identified by the glass-box approach of this study.

### Artificial intelligence for exploring and searching for rules

Finding transparent link functions (LFs) is done by the Bayesian evolution algorithm on the ground of the so-called “glass-box” rule-learning, which has been successfully applied to other complex physics phenomena suffering from the absence of first principles across wide length scales—the nanoscale tribo-charging^32^, the millimeter-scale wet-to-dry bubble transition^33^, the centimeter-scale composite heterogeneous materials^34,35^, and the extreme failures in earth lithosphere^36^. Detailed descriptions of the LFs and the glass-box rule-learning are presented in Method and Supplementary Information. To use the LF (denoted $\mathcal{L}$), it is efficient to normalize the input arguments. Based on the experimental setup, the UV dose is normalized as $\overline{{U }_{v}}={U}_{v}/4000 [\mathrm{J}/{\mathrm{m}}^{2}]\in {\mathbb{R}}[\mathrm{0,1})$. In this study, the relations between the UV dose and the physics terms $\sigma$ and $\mu$ will be related by a flexible and expressive function as $\sigma \left({U}_{v}\right)={\sigma }_{0}{\mathcal{L}}_{\sigma }\left(\overline{{U }_{v}};{{\varvec{\uptheta}}}_{\sigma }\right)$ and $\mu \left({U}_{v}\right)={\mu }_{0}{\mathcal{L}}_{\mu }\left(\overline{{U }_{v}};{{\varvec{\uptheta}}}_{\mu }\right)$ where ${\sigma }_{0}$ and ${\mu }_{0}$ are the surface tension and the dynamic viscosity of the liquid in the absence of the UV dose. Herein, ${\varvec{\uptheta}}$ denotes the free parameter vector to be learned and evolved by the adopted machine learning method (see Methods). This paper assumed ${\sigma }_{0}=0.04 \, \mathrm{N}/\mathrm{m}$ and ${\mu }_{0}=0.4 \, \mathrm{N s}/{\mathrm{m}}^{2}$, and there is no restriction to the use of other initial values. ${D}_{K}\left(\frac{d\overline{m} }{d\overline{t} }\right)={{D}_{K0}\mathcal{L}}_{{D}_{K}}\left(\underset{\forall t}{\mathrm{max}}\left[\frac{d\overline{m} }{d\overline{t} }\right];{{\varvec{\uptheta}}}_{{D}_{K}}\right)$ where ${D}_{K0}$ is the initial value of ${D}_{d}{K}_{H}$, and this study assumed ${D}_{d}{K}_{H}=1\times {10}^{-12}.$ This LF takes the all-time maximum mass flux rate to realize the common sense-based assumption that the diffusivity tends to irreversibly decrease once the nanopores are clogged by air molecules (Fig. 3c). The irreversible decrease of diffusivity may be released and sophisticated by accounting for the nanopore’s structural changes in terms of air pressure, increasing PDMS molecular strains, enlarged pore diameter, pore paths, and so on, which would be addressed in the future extensions of this paper.

To successfully design and devise a link function of the hidden rule, it is important to retain generality and include basic observational knowledge. Assuming the hidden rules are completely arbitrary, we start from general forms of LFs, i.e., the cubic regression spline (CRS)-based LF as done in^32–36^. The CRS-based LFs can represent arbitrary nonlinear shapes by virtue of the flexibility of CRS^37,38^. But preliminary searching and learning with CRS showed poor prediction performance of the present problems, and thus it is rejected by hybrid intelligence framework. Within the hybrid intelligence framework, the human intelligence core adopts common senses from the basic physics, i.e., (1) dynamic viscosity increases with increasing UV dose used for curing NOA73 and (2) the larger mass flux rate and the smaller diffusivity of air into PDMS nano pores. With this basic knowledge, hybrid intelligence framework suggests a simple, yet general shape LF, i.e., two-parameter exponential LF^32–36^. It has a simple form as $\mathcal{L}\left(x\right)=\mathrm{exp}({c}_{1}{x}^{{c}_{2}})$, but it can reproduce general nonlinear increasing/decreasing relations with only two parameters (Fig. S3).

## Discussion

### Data-driven identification of nonlinear dependence of surface tension on UV dose

**Figure 4:**
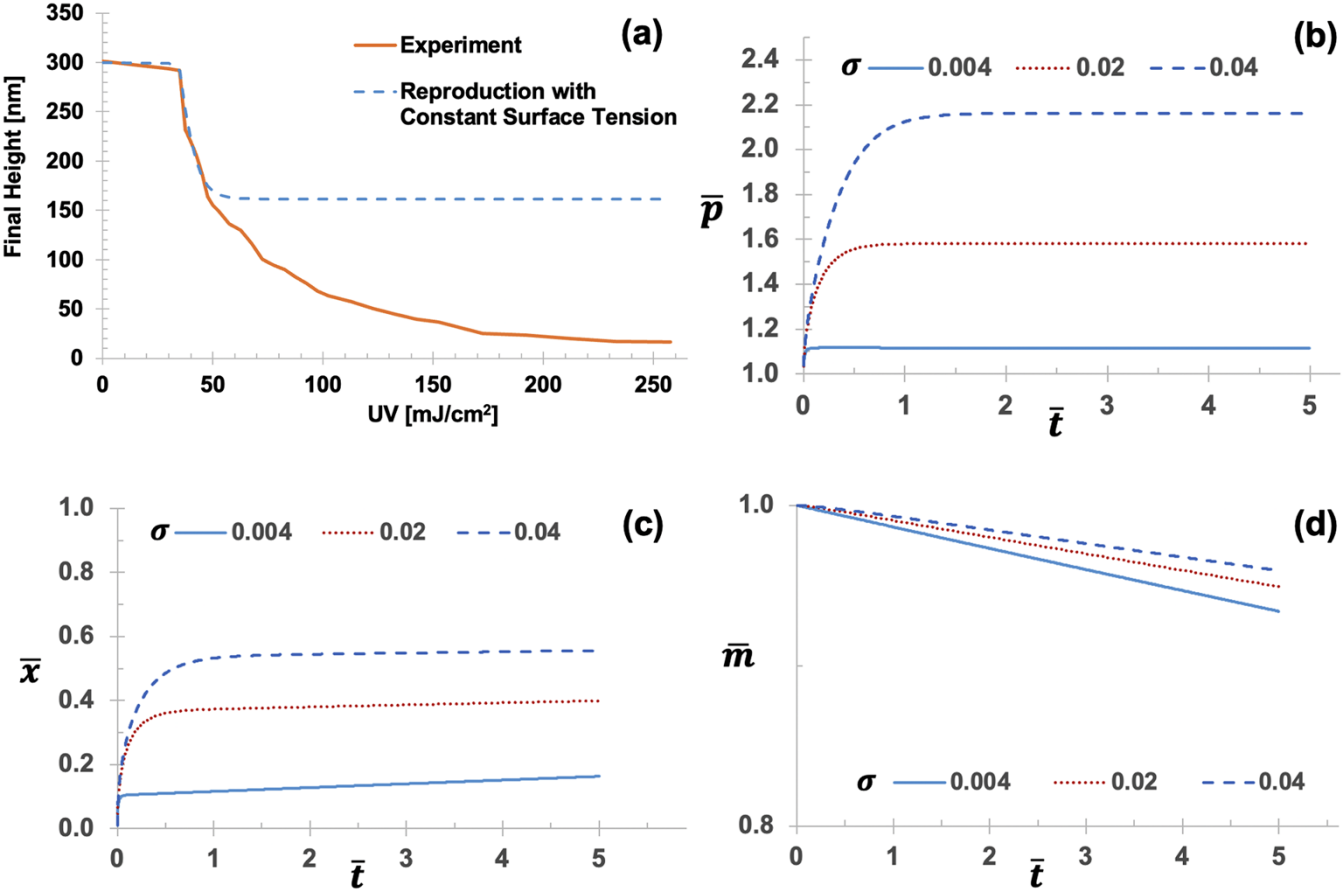
Role of the constant surface tension. (**a**) Poor performance in reproducing nanocapillary rise with two best-so-far LFs of dynamic viscosity and diffusivity along with the constant surface tension. Due to the large errors in the regime of high UV doses, this prediction rule is rejected by the hybrid intelligence framework. Even if both diffusivity and dynamic viscosity are learned as nonlinear relationships via corresponding LFs, the constant surface tension-based rules performed poorly. (**b**–**d**) Parametric study of the impact of the surface tension $\sigma$ on (**b**) normalized liquid height, (**c**) normalized air mass, and (**d**) normalized air pressure. Other parameters are fixed, the diffusivity ${{D}_{d}K}_{H}=1\times {10}^{-13}$ [mol s/kg] and the dynamic viscosity $\mu =0.4\text{ N s/}{\text{m}}^{2}$.

The surface tension of general liquids stems from the effects of intermolecular forces at the liquid–gas interface. In general, the surface tension of a liquid is regarded to decrease with increasing temperature since the increase in temperature accelerates the molecular thermal activity, thus decreasing the cohesive forces. The data-driven search for hidden rules via the hybrid intelligence approach appears to unravel such temperature-dependent surface tension of NOA73 without pre-defined adoption of an empirical rule (e.g.^39^) or analytical formulations. In this study, the UV exposure used for pre-curing of NOA73 may be the source of the temperature increase. During the rule-learning process of hybrid intelligence, any prediction rules with constant surface tension (i.e., independent of UV doses) were identified as unsuccessful and thus rejected. As shown in Fig. 4, the surface tension appears to govern the ultimate rising height, i.e., how high the capillary rising could continue is proportional to the surface tension, $\overline{x }\left(t\to \infty \right) \propto \sigma$. Although both the diffusivity and dynamic viscosity were learned as nonlinear relationships via LFs, without the UV-dependent (i.e., temperature-dependent) nonlinear relationship of the surface tension, the overall performances were poor (Fig. 4a). Thus, the rules with constant surface tension were rejected by the hybrid intelligence framework.

### Equivalent roles of diffusivity and dynamic pressure on nanocavity filling

Figure S4 shows the results of the parametric study which investigated the impact of the air diffusivity. The larger the product of ${{D}_{d}K}_{H}$, the faster the liquid rise (Fig. S4). At an extremely low diffusivity (e.g., ${{D}_{d}K}_{H}\sim {10}^{-13})$, the air escape becomes negligibly slow, and thus the liquid rising nearly stops. By comparing Figs. S4 and S5, the quantitative impacts of both dynamic viscosity and diffusivity on the final capillary rising height, mass reduction, and pressure increase appear to be nearly identical, i.e., the capillary rising height is proportional to the diffusivity, $\overline{x }\left(t\to \infty \right) \propto {{D}_{d}K}_{H}$ and to the dynamic viscosity, $\overline{x }\left(t\to \infty \right) \propto \mu$. The overall height change due to a 100-fold increase in $\mu$ (Fig. S4) is nearly identical to the cases in which ${D}_{d}{K}_{H}$ increases by a 100-fold (Fig. S5). This is, in fact, an anticipated behavior. Recall that $\beta$ of Eq. (2) can be rewritten as

3 $$\beta =\frac{2\mu {D}_{K}RT}{r\sigma \mathrm{cos}(\theta )}\frac{L}{{L}_{cr}}$$

We can confirm $\beta \propto \mu \left({U}_{v}\right)$ and $\beta \propto {D}_{K}\left(\frac{d\overline{m} }{d\overline{t} }\right)$ simultaneously. Since $\frac{d\overline{m} }{d\overline{t} }\propto \beta$, the comparable roles of the dynamic viscosity and diffusivity manifest clearly in the mass reduction and height rises, as commonly seen in Figs. S4 and S5.

In conclusion, this paper proposes a hybrid intelligence framework in which human’s insights and machine’s computing power can play in concert to unravel the hidden rules from one unexplained phenomenon in nanoscale capillary force lithography—the appearance of a “forbidden gap” in the capillary rise. The proposed framework helps overcome the lack of large data and the absence of first principles underlying the nanoscale phenomenon. Three rules are unraveled by the hybrid intelligence: the diffusivity as a nonlinear function of the air mass flux rate into porous media, the dynamic viscosity, and the surface tension of photopolymer as a function of the UV doses. The uncertainty of the identified rules is estimated. It can be reduced by future Bayesian rule learning which can smoothly incorporate more data and further physical insights. The future extension will be straightforward since the hybrid intelligence framework is built upon a transparent, physics-retained rule learning core as well as a Bayesian evolutionary algorithm, rendering itself suitable for expansion and evolution. This study promotes a synergistic nexus where scientists and artificial intelligence work together to push the unexplored boundaries of nanotechnologies and beyond.

## Methods

### Experiment procedure for nano capillary rising tests

To examine the nano-capillary rising behavior, a mold with nano cylindrical array was firstly prepared from the silicon master. The silicon master (LightSmyth) was spin-coated with the hard poly(dimethylsiloxane) (*h*-PDMS) mixture^17^ for 15 s at 500 rpm and 45 s at 1000 rpm. Subsequently, the spin-coated silicon master was placed on the hotplate 20 min at 60 °C. Then, a separately prepared soft PDMS (base and curing agent weight ratio = 10:1, Sylgard 184, Dow Corning) was poured onto the partially cured *h*-PDMS layer followed by the curing for 12 h at 60 °C. Once the mold was completely cured, it was peeled off from the silicon master. After the completion of the *h*-PDMS mold preparation, NOA73 (Norland Inc.), a photocurable polymer, was spin-coated on a glass substrate for 15 s at 550 rpm and then 45 s at 3000 rpm. The spin-coated photocurable polymer was exposed to the collimated UV LED (M365LP1-C2, Thorlabs) modulated by digital micromirror device (DMD, DLP 7000UV, Texas Instruments). For the height-dose curves, the intensity of UV LED was set to 2.5 mW/cm^2^ and the exposure time was controlled through the pre-programmed DMD sequence. The UV-exposed photopolymer thin film was covered with the *h*-PDMS mold for 2 min. Subsequently, it was exposed to the UV light for an additional curing 90 s (30 mJ/cm^2^). Finally, the *h*-PDMS mold was peeled off to reveal the nanopillar arrays.

### Data preparation from nano capillary experiments

The topography of the printed nanopillars array was measured by atomic force microscopy (AFM) in the tapping mode (Multimode, Veeco) using highly doped silicon tips (NCHR, NanoWorld) with the resonance frequency of 320 kHz, and the spring constant 42 N/m. The number of data points in columns and rows both were set to be 256 points. The scan size and rate were chosen to 3 µm × 3 µm and 0.5 Hz/s, respectively. From the resulting AFM images, the data of each sample was extracted in a text file by utilizing NanoScope Analysis v1.90 (Veeco). All experimental data will be made publicly available upon request to the authors.

### Force equilibrium formulation

Phan et al.^18^’s analytical derivation of fluid filling process in a closed end nanochannel serves as the base of this paper’s formulations of force equilibrium and mass balance. Clear novelty of this paper lies in the facts that the nonlinear diffusivity of air molecules to the porous PDMS media is incorporated herein, and several unknown hidden rules are primary target of learning by hybrid intelligence. In general, the rise and stop of the fluid inside a vertical, prismatic cylinder nanocavity is governed by four force terms: ${F}_{s}-{F}_{v}-{F}_{P}-{F}_{g}=0$ where ${F}_{s}$ is the capillary force driven by the surface tension of the liquid; ${F}_{v}$ is the viscous force dragging down the rise of liquid; ${F}_{P}$ is the pressure of air confined in the nanocavity; ${F}_{g}$ is the gravitational force. As depicted in Fig. 3a,b, if the nanopillar has a circular cross-section with radius $r$ and height $L$ and the rise height of the liquid is $x(t)$ at time $t$, the force terms are ${F}_{s}=\left(2\pi r\right)\cdot \sigma \left({U}_{v}\right)\cdot \mathrm{cos}\left(\theta \right);{F}_{v}=\left(2\pi r\right)\cdot x\left(t\right)\cdot \left(\frac{4\mu ({U}_{v})}{r}\frac{dx}{dt}\right);{F}_{p}=\left(\pi {r}^{2}\right)\cdot \left(p\left(t\right)-{p}_{0}\right);{F}_{g}=\left(\pi {r}^{2}x(t)\right)\cdot {\gamma }_{l}\cdot g$ where $\sigma ({U}_{v})$ [N/m] is the surface tension of the liquid which, in turn, depends on the UV dose ${U}_{v} [\mathrm{J}/{\mathrm{m}}^{2}]$; $\theta$ [deg] is the contact angle between the liquid and the nano cavity (75 degree); $\mu \left({U}_{v}\right) [\frac{\mathrm{N}}{{\mathrm{m}}^{2}}\mathrm{ s}]$ is the ${U}_{v}$-dependent dynamic viscosity of the liquid; $\frac{dx}{dt}$ is the average velocity of the rise of the liquid; $\frac{4\mu }{r}\frac{dx}{dt}$ is the wall shear stress of a cylinder where the flow velocity profile is assumed parabolic; $p\left(t\right) [\frac{\mathrm{N}}{{\mathrm{m}}^{2}}]$ is the pressure at time $t$ of the confined air; ${p}_{0}$, the initial air pressure, is assumed to be the atmospheric pressure; ${\gamma }_{l}$ is the density of the liquid; $g$ is the gravity acceleration.

Plugging all force terms into the equilibrium equation leads to $2\pi r\sigma ({U}_{v})\mathrm{cos}\left(\theta \right)-2\pi rx\left(t\right)\left(\frac{4\mu \left({U}_{v}\right)}{r}\frac{dx}{dt}\right)-\pi {r}^{2}\left(p\left(t\right)-{p}_{0}\right)-\pi {r}^{2}x(t){\gamma }_{l}g=0$ As the gravity force is negligibly smaller than other terms (e.g., ${F}_{s}/{F}_{g}>{10}^{9}$ for water in the nano cavity dimensions of this paper), we can ignore the last term hereafter. By applying the ideal gas law to the confined air, the pressure terms are given as $p\left(t\right)=\frac{m\left(t\right)RT}{M\pi {r}^{2}(L-x\left(t\right))}; {p}_{0}=\frac{m\left(0\right)RT}{M\pi {r}^{2}L}$ where $m\left(t\right)$ [kg] is the mass at time $t$ of the confined air; $R$ the universal gas constant; $T$ [K] the absolute temperature (78 K); $M$ [kg/mol] the molar mass of the confined air (0.02897 kg/mol). Substituting the pressure terms into the equilibrium, we obtain the liquid rise velocity equation

4 $$\frac{dx}{dt}=\frac{r\sigma ({U}_{v})\mathrm{cos}\left(\theta \right)}{4\mu ({U}_{v})x\left(t\right)}-\frac{RT}{8\pi M\mu \left({U}_{v}\right)x\left(t\right)}\left(\frac{m\left(t\right)}{L-x\left(t\right)}-\frac{m\left(0\right)}{L}\right).$$

### Mass balance formulation

The air confined in the nanocavity will decrease through diffusion into the nanopores in the PDMS mold. By the Fick’s law the mass balance of the confined air can be written as $\frac{dm}{dt}=-\frac{{D}_{d}M}{{L}_{cr}}{{A}_{nc}(t) (K}_{H}p\left(t\right)-{K}_{H}{p}_{0})$, where ${A}_{nc}\left(t\right)$ is the surface area of the nanocavity over which air can diffuse into the PDMS bulk, ${A}_{nc}\left(t\right)=\left(\pi {r}^{2}+2\pi r\left(L-x\left(t\right)\right)\right);{K}_{H} \left[\frac{\mathrm{mol}}{{\mathrm{m}}^{3}\mathrm{Pa}}\right]$ is the Henry constant determining the concentration of air molecules; ${D}_{d} [\frac{{\mathrm{m}}^{2}}{\mathrm{s}}]$ is the diffusion coefficient of the air into the PDMS mold which is modeled as a porous air-permeable medium^40,41^; ${L}_{cr}$ is the nanopore pressure critical length [m] measured from the nanocavity’s wall. At ${L}_{cr}$, the internal pore pressure becomes ${p}_{0}$ (Fig. 3). ${L}_{cr}$ may be dependent upon various internal nanoporous media properties including the porosity, tortuosity, and mean pore diameter^42^. In this work, ${L}_{cr}$ is assumed to be constant. For a better understanding of the physical meaning of ${L}_{cr}$, Fig. S6 shows the impacts of ${L}_{cr}$ on the nanocapillary rise, air mass reduction, and air pressure change as ${L}_{cr}$ is varied between $L$ and $10L$. A larger ${L}_{cr}$ physically implies a larger zone of nanopores in the PDMS mold. Thus, the mass reduction and capillary rise become slower as ${L}_{cr}$ increases. In this study, our transparent ML method focuses on unraveling the rules for UV dose-dependent nonlinear diffusivity and surface tension. To facilitate the ML process, we fixed ${L}_{cr}=10L$. In reality, the nanoporous zone in PDMS may vary with the pressure and air velocity^42^. Further investigations into such nonlinear impacts of ${L}_{cr}$ shall be a future extension of this study. By substituting the pressure terms into the mass balance, we obtain

5 $$\frac{dm}{dt}=-\frac{{D}_{d}{K}_{H}RT{A}_{nc}\left(t\right)}{{L}_{cr}\pi {r}^{2}L\left(L-x\left(t\right)\right)}\left(m\left(t\right)L-m\left(0\right)\left(L-x\left(t\right)\right)\right).$$

### Bayesian evolutionary algorithm

To realize the smooth evolution of the rule-learning, this paper adopted a combination of the fitness-proportionate probability (FPP) rule of the genetic algorithm and the Bayesian update scheme. According to FPP rule, the probability that an organism $s$ (i.e., unique realization of all hidden rules’ free parameters ${\varvec{\Theta}}=\{{{\varvec{\theta}}}_{1},\boldsymbol{ }\dots ,\boldsymbol{ }{{\varvec{\theta}}}_{{n}_{rule}}\}$, ${n}_{rule}$ = number of total hidden rules) in the current generation is selected for the next generation is proportional to the fitness score ($\mathcal{F}$),

$\mathcal{F}\left(s\right)={\left(1+Err\left(s\right)\right)}^{-1}$ where $Err(s)={n}^{-1}{\sum }_{i}^{n}{(x}_{real}^{(i)}-{x}_{pred}^{\left(i\right)}{)}^{2}/{(x}_{real}^{\left(i\right)}{)}^{2}.$ The smaller error, the fitter. The prior best generation’s fitness scores and the set of all ${\varvec{\Theta}}$ of the prior best generation are denoted by ${\mathcal{F}}^{*}(s)$ and ${S}^{\mathbf{*}}({\varvec{\Theta}})$, respectively. As $s$ can uniquely realize each ${\varvec{\Theta}}$, $s$ and ${\varvec{\Theta}}$ are interchangeable: $p\left(s\right)\propto \mathcal{F}\left(s\right)$, equivalently $p\left({\varvec{\Theta}}\right)\propto \mathcal{F}\left(s\right).$ Bayesian fitness score of an individual new organism (${\mathcal{F}}_{B}\left(s\right)$) is then defined as ${\mathcal{F}}_{B}(s)=\frac{1}{\kappa }\frac{\mathcal{F}(s; {S}^{*}\left({\varvec{\Theta}}\right)){\mathcal{F}}^{*}(s)}{{\sum }_{\forall s}{\mathcal{F}}^{*}(s)}$ where normalization term $\kappa ={\sum }_{\forall s}\frac{\mathcal{F}(s; {S}^{*}\left({\varvec{\Theta}}\right)){\mathcal{F}}^{*}(s)}{{\sum }_{\forall s}{\mathcal{F}}^{*}(s)}.$ Then, next generation’s parent selection probability is $p\left({\mathrm{parent}}_{i}| s\right)\propto {\mathcal{F}}_{B}\left(s\right), \left(i=\mathrm{1,2}\right)$. Therefore, all the identified LFs (via ${\varvec{\Theta}}$) can smoothly evolve with new experimental data. On the Bayesian evolutionary framework, in total 100,000 organisms and 10 generations are used, 4 alleles are used for each gene, and the variable-wise mutation scheme is used with the mutation rate of 0.005. Hybrid intelligence framework suggests the best-performing search ranges ${c}_{1}\in \left[-4, 0\right]$ and ${c}_{2}\in \left[0, 10\right]$ for LFs of diffusivity and surface tension whereas ${c}_{1}\in \left[0, 4\right]$ and ${c}_{2}\in \left[0, 10\right]$ for LF of dynamic viscosity. More detailed explanations and formulations of the Bayesian evolutionary algorithm are available in^32–34^.

### Supplementary Information

Supplementary Information.

## Data Availability

The datasets generated and analyzed during the current study are available in the cloud-based data repository, [https://iastate.box.com/s/qai02n2od3ctxw32cxm10y29k8hl68me].
