# Supplementary material for: Pursuit of hidden rules behind the irregularity of nano capillary lithography by hybrid intelligence
Source: Sci Rep. 2023 Aug 22;13:13649. doi: 10.1038/s41598-023-41022-7 (PMC10444899; doi:10.1038/s41598-023-41022-7)
Supplement: Supplementary file 1 — Supplementary Information. [file 41598_2023_41022_MOESM1_ESM.pdf]

Supplementary Information

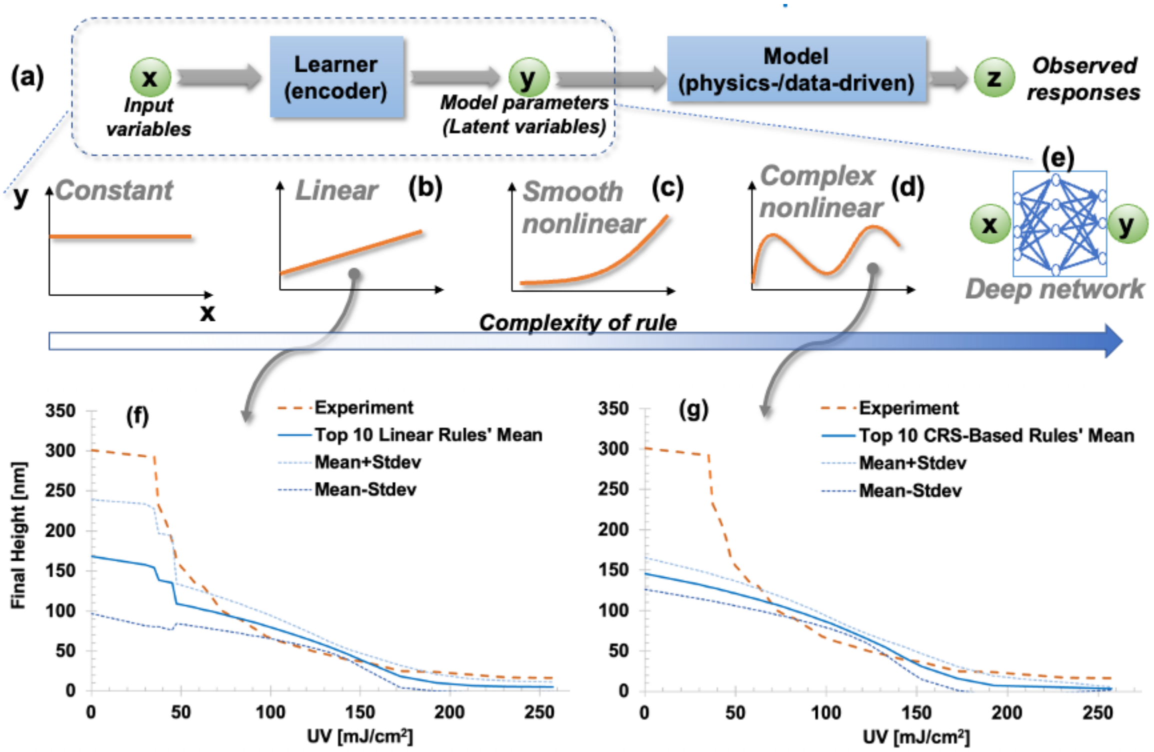

Fig. S1. Illustration of the three global stages of the rule learning with diverse candidates for the hidden rules of the salient parameters ($y \in \{D_k, \mu, \sigma\}$) in terms of descriptive variables ($x \in \{d\bar{m}/d\bar{t}, \overline{U_v}\}$): (a) Overall learning architecture from input x to latent variables y (internal parameters), and to the observed responses z (e.g., the final height of nano capillary rise); (b) Linear model-based rule; (c) Smooth monotonic curve-based rule; (d) Complex nonlinear curve-based rule that can be learned by the cubic regression splines (CRS); (e) Deep neural network-based rule for the most general (yet black-box) rule; (f-g) Examples of the best-so-far predictions that use the linear model-based rules (f) and CRS-based rules (g), both exhibiting poor performance.

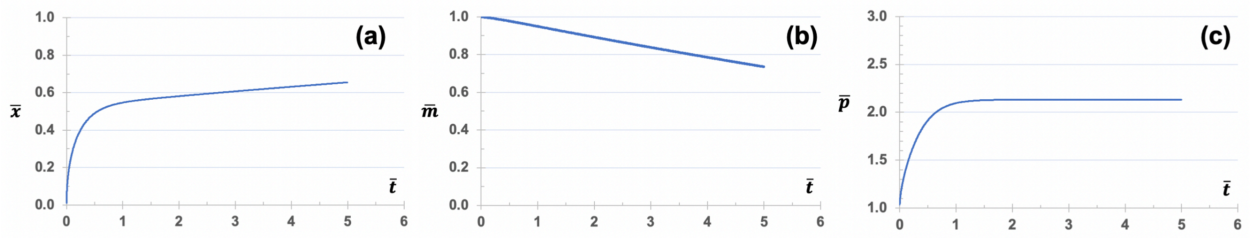

Fig. S2. Basic evolution of liquid and air under constant diffusivity, surface tension, and dynamic viscosity: (a) Normalized liquid height continues to increase to approach the full nanocavity height ($\bar{x} \to 1.0$); (b) Normalized air mass continues to decrease by constant air diffusion into PDMS nanopores ($\bar{m} \to 0.0$); (c) Normalized air pressure reaches stability as the air volume reduction due to the liquid rise becomes balanced by the air mass reduction.

Fig. S2 shows that under the constant diffusivity of air, the liquid eventually reaches the full height of the nanocavity since the confined air finds stability between the reduced volume and reduced mass. If there is no change in diffusivity, the liquid should be able to fill in the entire nanocavity. As long as the air diffusivity does not change, the air eventually diffused away into the PDMS nanopores, and the liquid can always reach the full height of the nanocavity.

Table S1. The best-so-far free parameters of three link functions used for Fig. 2(a)

Link function	$\boldsymbol{\theta} = \{c_1, c_2\}$	Best search range	Description
$\mathcal{L}_{DK}(\frac{d\bar{m}}{d\bar{t}}; \boldsymbol{\theta}_{DK})$	$\boldsymbol{\theta}_{DK} = \{-2.63529, 0.62745\}$	$c_1 \in [-4, 0], c_2 \in [0, 10]$	Diffusivity
$\mathcal{L}_{\mu}(\overline{U_v}; \boldsymbol{\theta}_{\mu})$	$\boldsymbol{\theta}_{\mu} = \{3.09020, 0.07843\}$	$c_1 \in [0, 4], c_2 \in [0, 10]$	Dynamic viscosity
$\mathcal{L}_{\sigma}(\overline{U_v}; \boldsymbol{\theta}_{\sigma})$	$\boldsymbol{\theta}_{\sigma} = \{-3.26275, 0.23529\}$	$c_1 \in [-4, 0], c_2 \in [0, 10]$	Surface tension

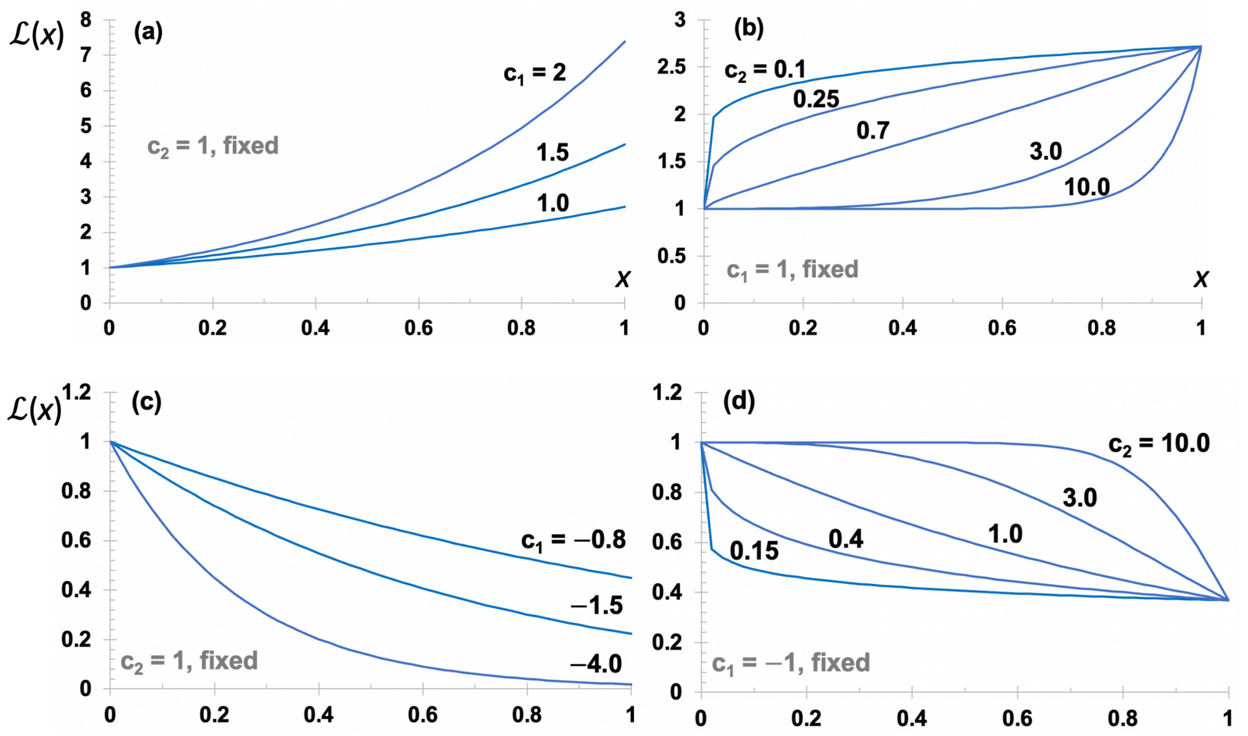

Fig. S3. Example plots of the two-parameter exponential link function: General nonlinear increasing relations (a-b) and decreasing relations (c-d) where $\mathcal{L}(x) = \exp(c_1 x^{c_2})$.

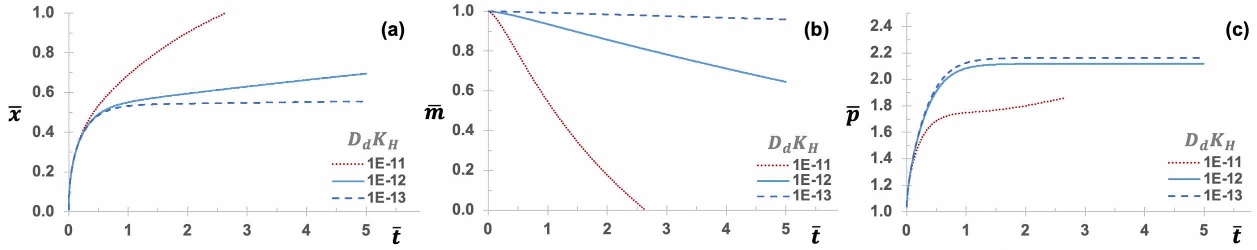

Fig. S4. Parametric study of impact of the air diffusivity $D_d K_H$ [mol.s/kg]**:** (a) Normalized liquid height; (b) Normalized air mass; (c) Normalized air pressure. Other parameters are fixed, the dynamic viscosity $\mu = 0.4\ \mathrm{N.s/m^2}$ and the surface tension $\sigma = 0.04\ \mathrm{N/m}$.

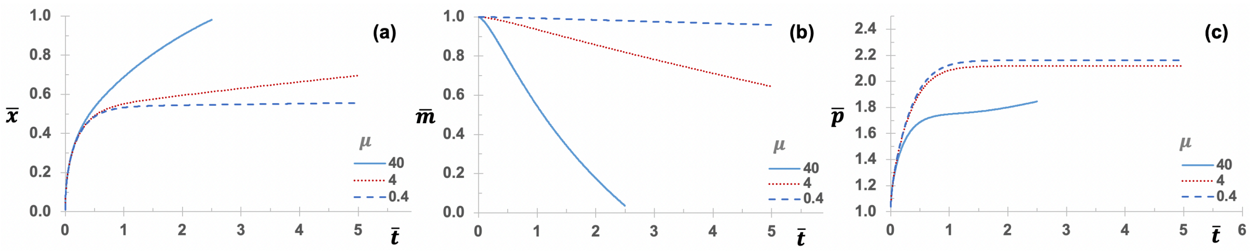

Fig. S5. Parametric study of impact of the dynamic viscosity μ $[\mathrm{N.s/m^2}]$: (a) Normalized liquid height; (b) Normalized air mass; (c) Normalized air pressure. Other parameters are fixed, the diffusivity $D_d K_H = 1 \times 10^{-13}$ [mol.s/kg] and the surface tension $\sigma = 0.04$ N/m.

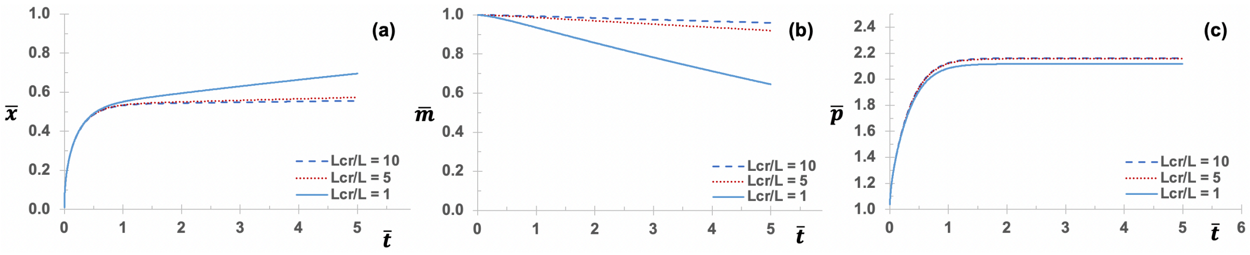

Fig. S6. Parametric study of the impact of the nanopore pressure critical length L_{cr} on: (a) Normalized liquid rise height; (b) Speed of normalized air mass reduction; (c) Normalized air pressure.

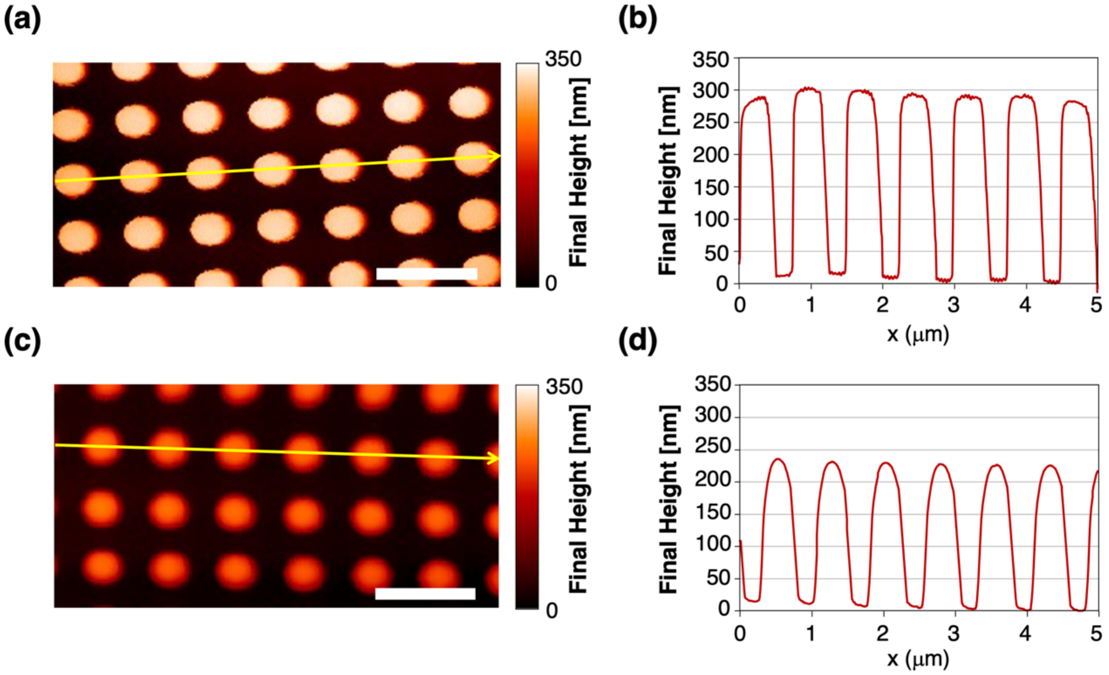

Fig. S7. Atomic force microscopy (AFM) scans of the nanopillar array near the forbidden gap (UV dose – near 35 mJ/cm^2): (a) AFM image right above the forbidden gap with h = 292 nm; (b) The final height profile taken along the yellow arrow scan lines in (a); (c) AFM image right below the forbidden gap with h = 232 nm; (d) The final height profile taken along the yellow scan lines in (c). (Scale bars: 1 μm).
